# TGFβ level in healthy and children with Marfan syndrome—effective reduction under sartan therapy

**DOI:** 10.3389/fped.2024.1276215

**Published:** 2024-02-05

**Authors:** Veronika C. Stark, Jakob Olfe, Daniel Diaz-Gil, Yskert von Kodolitsch, Rainer Kozlik-Feldmann, Johannes Reincke, Maria Stark, Peter Wiegand, Tanja Zeller, Thomas S. Mir

**Affiliations:** ^1^ Clinic for Pediatric Heart Medicine and Adults with Congenital Heart Disease, University Heart and Vascular Center, University Medical Center Hamburg-Eppendorf, Hamburg, Germany; ^2^ Department of Cardiac Surgery & Department of Pediatric, Boston Children’s Hospital/ Harvard Medical School, Boston, MA, United States; ^3^ Department of Cardiology, University Heart and Vascular Center, University Medical Center Hamburg-Eppendorf, Hamburg, Germany; ^4^ Institute of Medical Biometry and Epidemiology, University Medical Center Hamburg-Eppendorf, Hamburg, Germany; ^5^ Department of General and Interventional Cardiology, University Center of Cardiovascular Science, University Heart and Vascular Center, University Medical Center Hamburg-Eppendorf, Hamburg, Germany; ^6^ Deutsches Zentrum für Herzkreislaufforschung, Hamburg, Germany

**Keywords:** Marfan, TGFβ, sartan, connective tissue disorder, genetic aortic disease, aortic dilatation

## Abstract

**Introduction:**

Transforming growth factor β (TGFβ) metabolism plays an important role in the pathogenesis of Marfan syndrome (MFS). Accordingly, drug therapy uses TGFβ receptor blockade to slow down the cardiovascular manifestations, above all aortic root dilatation. Angiotensin II type 1 receptor blockers (ARBs) have been shown to reduce TGFβ levels in adults. Data on childhood are lacking and are now being investigated in the TiGer For Kids study presented here.

**Methods:**

We examined 125 children without chronic disease and 31 pediatric Marfan patients with a proven *FBN1* variant with regard to TGFβ levels. In addition, we measured TGFβ levels during the initiation of ARB therapy in pediatric Marfan patients.

**Results:**

In children without chronic disease, TGFβ levels were found to decrease from childhood to adolescence (*p* < 0.0125). We could not measure a relevantly increased TGFβ level in pediatric Marfan patients. However, we showed a significant suppression of the TGFβ level after treatment with ARBs (*p* < 0.0125) and a renewed increase shortly before the next dose.

**Discussion:**

The TGFβ level in childhood changes in an age-dependent manner and decreases with age. The TGFβ level drops significantly after taking ARBs. Based on our experience and data, a TGFβ receptor blockade in childhood seems reasonable. So far, TGFβ level cannot be used as an MFS screening biomarker.

## Introduction

Marfan syndrome (MFS) is classically caused by a pathogenic fibrillin-1 (*FBN1)* variant (1, 2). The dysfunction of fibrillin-1 itself as an extracellular matrix protein associated with microfibrils was, for a long time, known to be the only cause for this structural weakness of connective tissue in MFS. However, in 2003, Neptune et al. showed a function of fibrillin-1 for the regulation of transforming growth factor β (TGFβ). A pathogenic variant in fibrillin-1 results in an enhancement of TGFβ, whereas TGFβ activation then prohibits regeneration of vascular smooth muscle cells in, for example, the aortic wall (3). Accordingly, both the altered fibrillin structure itself and the dysregulation of TGFβ cause dilatation of the aortic root, and other cardiovascular, skeletal, skin, and ocular manifestations appear.

Due to this exceeding activation of TGFβ in MFS, medical therapy with angiotensin II type 1 receptor blockers (ARBs), also known as sartans, was expected to be highly effective and started in patients with MFS (4). Blockade of angiotensin II type 1 receptor causes the downregulation of TGFβ and stimulates the regeneration of vascular smooth muscle cells with protection of connective tissue, especially in the aortic wall. Aortic root dilatation could thus be prevented (5). ARBs are therefore, apart from β-adrenergic receptor antagonists (β-blockers), which have been the only cardiovascular medical treatment for MFS patients for a long time, a new option (6). The positive correlation of TGFβ dysregulation with the appearance of cardiovascular pathologies in MFS and other genetic aortic syndromes was already shown in adult patients (7, 8). The decrease of TGFβ concentrations after initiation of therapy could also be shown (9). Moreover, Franken et al. showed that therapy with sartans (losartan) leads to a significant decrease in aortic diameters in adult patients with haploinsufficient *FBN1* variants. This was not demonstrated in the group with dominant-negative gene variants (10). Corresponding data concerning sartan treatment in childhood are still lacking. Especially, measurements of TGFβ levels in MFS children with ARB therapy are missing but essential to evaluate the effectiveness of this therapy. It may be reasonable to initiate therapy in early childhood before the appearance of, especially, cardiovascular pathologies to prevent symptoms of MFS (11).

The aim of this prospective study is to evaluate the serum TGFβ level in children without chronic disease and in comparison to children with connective tissue disorder MFS with and without medical therapy. We investigate four primary research hypotheses: (1) TGFβ serum levels are higher in MFS patients than in healthy children. (2) TGFβ levels are higher in patients with MFS patients with aortic root dilatation than in healthy children. (3) TGFβ serum levels in patients with MFS reduce under medical treatment after 6 h. (4) Sinus valsalvae Z-scores in MFS patients reduce under medical treatment.

## Materials and methods

Our TiGer For Kids study is a monocentric prospective cohort study with the overall aim of comparing serum TGFβ levels in children without chronic disease with those of children with MFS and evaluating potential uses of TGFβ as a biomarker.

### Subjects

Between April 2017 and May 2020, we examined and obtained blood samples from children without chronic disease and pediatric Marfan patients with and without treatment. In the following, children without chronic disease are also referred to as healthy children.

We included healthy children whenever they presented to the emergency room or admitted to the pediatric ward with uncomplicated pediatric diseases (mild upper airway infection, gastroenteritis, commotio cerebri, motor and development disorders, and social and behavioral problems) without any cardiovascular disease or drugs, cancer, inflammatory or autoimmune disorder, diabetes, nor drugs that could affect the extracellular matrix. To exclude the influence of inflammation or renal impairment on the TGFβ level, we examine the difference of the TGFβ value between low and high c-reactive protein (CRP) and creatinine values of the patients with the TGFβ value.

We included children with MFS while visiting our specialized pediatric Marfan Clinic in the Pediatric Cardiology in the University Heart and Vascular Center Hamburg for first presentation or clinical follow-up. We diagnosed MFS according to the revised Ghent Criteria (12).

### Protocol: clinical examination and questionnaire

#### Baseline study

At the presentation of healthy children and pediatric Marfan patients, they received a standardized questionnaire about health and a blood sample. The questionnaire was used to precisely document and evaluate the patients’ previous medical history. The individual risk for a possible chronic, especially cardiovascular, disease was also recorded on the basis of family history. Blood sampling in non-chronically ill children was performed at the time when it was medically indicated. This can affect any time of the day or night. The TGFβ sample of Marfan patients was taken during Marfan consultation hours, mostly between 12 and 3 pm. Accordingly, there was no standardized time of measurement. In addition to the TGFβ level measurement, other laboratory parameters (CRP, creatinine) were analyzed to investigate their influence on the TGFβ level.

In addition, Marfan patients were examined according to the Ghent Criteria, and electrocardiogram (ECG) and echocardiography were performed. An experienced investigator performed echocardiography with the Philips diagnostic ultrasound system EPIQ7 (Amsterdam, Netherlands) with 12, 8, and 5 MHz probes.

#### Intervention study

**Figure 1:**
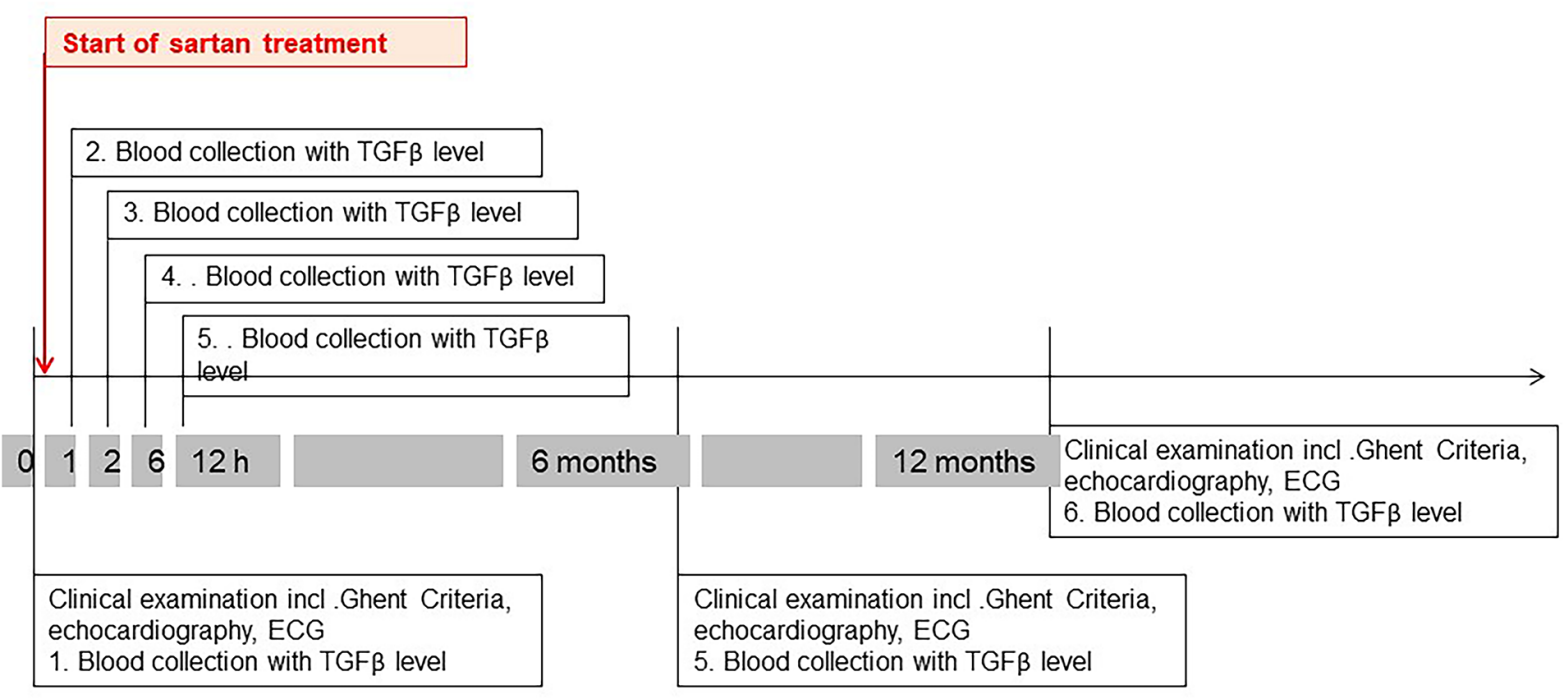
Timeline of care for Marfan patients with medical therapy.

As soon as medical therapy was indicated, we took a blood sample for measurements of TGFβ. Patients were admitted to the ward for 24 h. The first TGFβ measurement took place between 8 and 9 a.m. After we applied the ARBs, we repeated TGFβ measurement after 1, 2, 6, and 12 h. In our Marfan Clinic, we usually administer Valsartan two times a day. We start with a dose of approximately 1.5 mg/kg body weight daily and increase the therapy in the course of time. If therapy with losartan has already been started outside the clinic or patients favor losartan, we leave it at that. Thus, during the follow-up visit (6 and 12 months), we repeated the examinations from the baseline visit. The follow-up blood collection was also not standardized in terms of the time of day or in relation to the sartan intake. It was based on the time of presentation at the clinic. A timeline for the care of the MFS patients with medical treatment is shown in Figure 1.

### TGFβ measurements

Serum monovettes were centrifuged for 10 min at 3,000 g and 18°C. After filling and sealing of the tubes, the rack was stored directly in the working freezer at −80°C. Finally, TGFβ was determined in the laboratory by means of enzyme-linked immunosorbent assay (ELISA). For TGFβ measurement, we used the Quantikine human TGF 1 immunoassay (R&D Systems Inc. Minneapolis; catalogue number DB100B) according to the manufacturer's instructions. In order to avoid interfering variables, the TGFβ measurements were carried out simultaneously for all serum samples so that the experimental conditions in the laboratory were identical for all samples. The samples were measured in duplicate at points to ensure the accuracy of the values.

### Data analysis

In accordance with applicable data protection, the patients were pseudonymized, and a laboratory code and a study code were assigned to each child. The data were collected and archived in a database using Filemaker software V.10 Pro Advanced (Santa Clara, CA) and Microsoft Excel 2007 (Microsoft, Redmond, WA). We performed statistical analysis with SPSS 26 (SPSS, Chicago, IL).

We categorized patients into four age groups: infants (1 day- 24 months), children (25 months-11 years), adolescents (12–14 years), and adults (15–18 years).

In the descriptive statistics, we reported absolute numbers with relative frequencies for categorical data and mean values (M) ± standard deviation (SD) and quantiles for continuous data. All TGFβ values are given in pg/ml.

For analysis, TGFβ levels, CRP values, and creatinine values were approximated to a normal distribution using logarithmic transformation. The first and second primary hypothesis, as well as secondary comparisons in healthy children considering TGFβ levels between sex, low or high CRP, and low or high creatinine, were evaluated using unpaired *t*-tests. Furthermore, TGFβ levels were compared between age groups in healthy children using analysis of variance. The third and fourth primary hypotheses were analyzed using paired *t*-tests.

We adjusted the analysis results of the primary hypotheses using Bonferroni correction, which led to an adjusted significance level of 0.0125. All *p* values of the primary analysis, which were lower than the adjusted significance level, were considered to be statistically significant. We reported 95% confidence intervals and any results of secondary hypotheses in a descriptive way.

### Ethical standard

Informed and written consent was always obtained prior to blood sampling and testing by the doctors involved in the study. From the age of 6 years, in accordance with the recommendation of the Federal Court of Justice, the written consent of the child was obtained in addition to the verbal consent of the legal guardians. The study was approved by our Institutional Review Board [Ethics Committee Hamburg (PV 5457)].

## Results

We measured TGFβ levels in 125 children without chronic disease (9.4 ± 5.7 y; male 57.6%) and 31 pediatric Marfan patients (6.1 ± 5.6 y, male 51.6%) with a proven *FBN1* variant.

We present the age distribution of the healthy children and pediatric Marfan patients in Table 1. We demonstrate the clinical characteristics of the healthy and Marfan children in Table 1 and those of the Marfan patients with medical treatment in Table 2.

**Table 1:** Distribution of age and clinical characteristics of healthy children (control) and Marfan patients (MFS); ns, not significant.

Patient group	MFS (*n* = 31)	Control (*n* = 125)	** **
Age (years) at TGFβ measurement (mean ± SD)	6.1 ± 5.6	9.4 ± 5.7	
Age group (*n*, %)			
** **0–24 months	11 (35.5)	22 (17.6)	ns
** **2–11 years	14 (45.2)	43 (34.4)	ns
** **12–14 years	1 (3.2)	30 (24.0)	<0.05
** **15–18 years	5 (16.1)	30 (24.0)	ns
Sex (*n*, %)			ns
** **Female	15 (48.4)	53 (42.4)	
** **Male	16 (51.6)	72 (57.6)	
*FBN1* positive (*n*, %)	31 (100.0)	0 (0.0)	
Ghent criteria positive (*n*, %)	20 (64.5)	0 (0.0)	

**Table 2:** Clinical characteristics of Marfan patients with medical treatment (*n* = 11).

Age (years) at first TGFβ measurement (mean ± SD)	5.5 ± 3.8
Age group (*n*, %)	
0–24 months	4 (36.4)
2–11 years	6 (54.5)
12–14 years	1 (9.1)
15–18 years	0 (0.0)
Sex (*n*, %)	
Female	3 (27.3)
Male	8 (72.7)
*FBN1* positive (*n*, %)	11 (100.0)
Ghent criteria positive (*n*, %)	11 (100.0)

### TGFβ level in children without chronic disease

**Figure 2:**
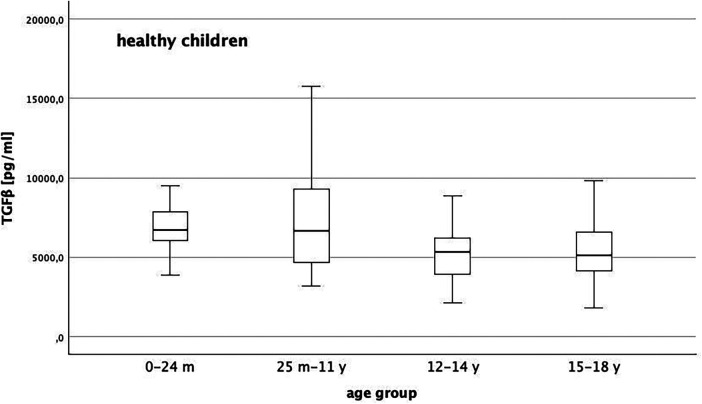
Distribution of TGFβ level in healthy children with a general decline of TGFβ the older the children get and lower levels with reach of puberty/adolescence (*p* = 0.001).

In children without chronic disease, median TGFβ level declines the older the children get (Figure 2). At the beginning of puberty and adolescence, TGFβ is lower than in younger children (*p* = 0.001). Concerning sex we did not identify any differences of TGFβ level at any age in children without connective tissue disorder (*p* = 0.87). Regarding the presence of inflammation (*p* = 0.83) or impaired kidney function (0.47), we could not show any influence on TGFβ levels in our studies.

### TGFβ level in pediatric Marfan patients

**Figure 3:**
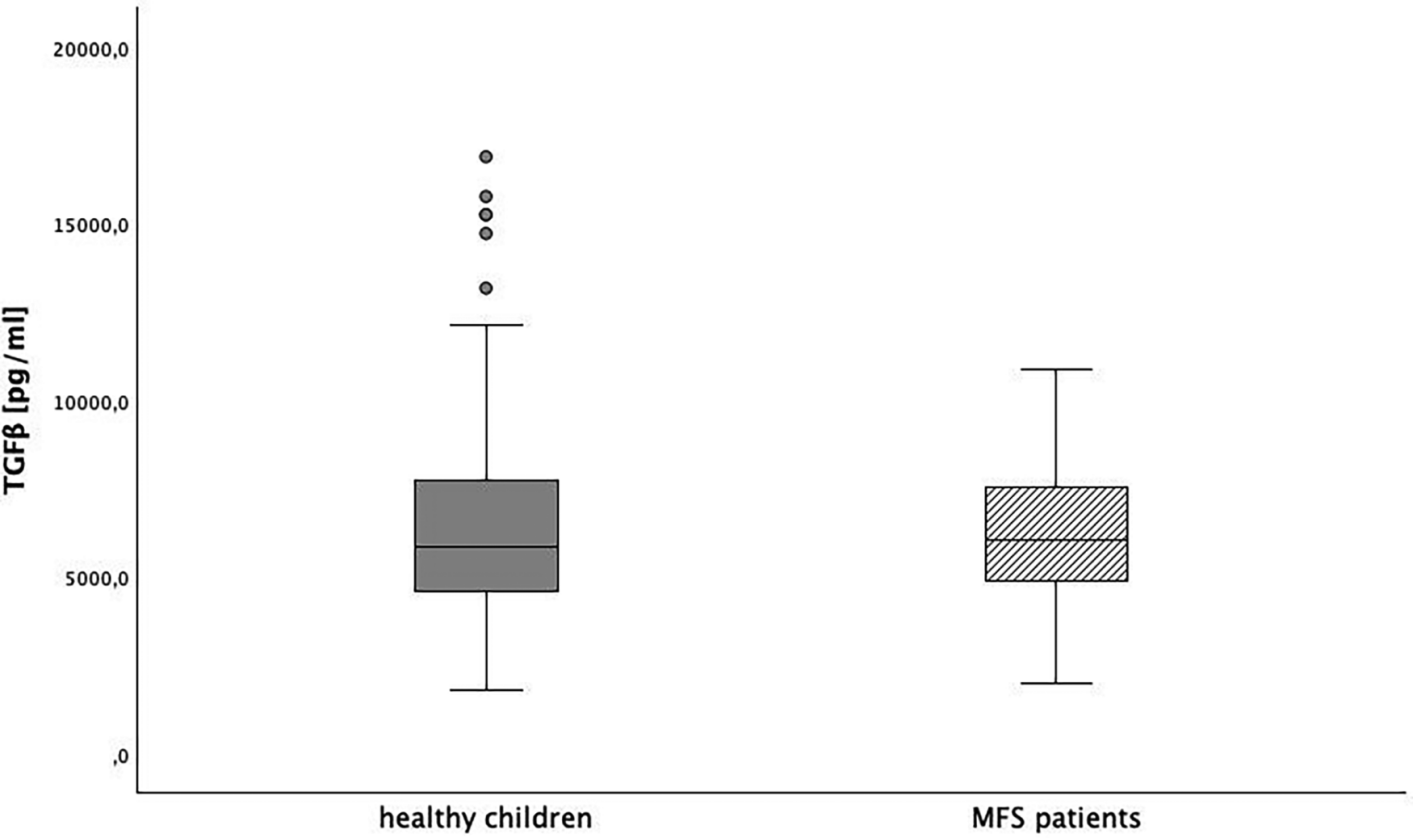
TGFβ level in healthy children and children with Marfan syndrome (MFS), *p* > 0.0125.

**Figure 4:**
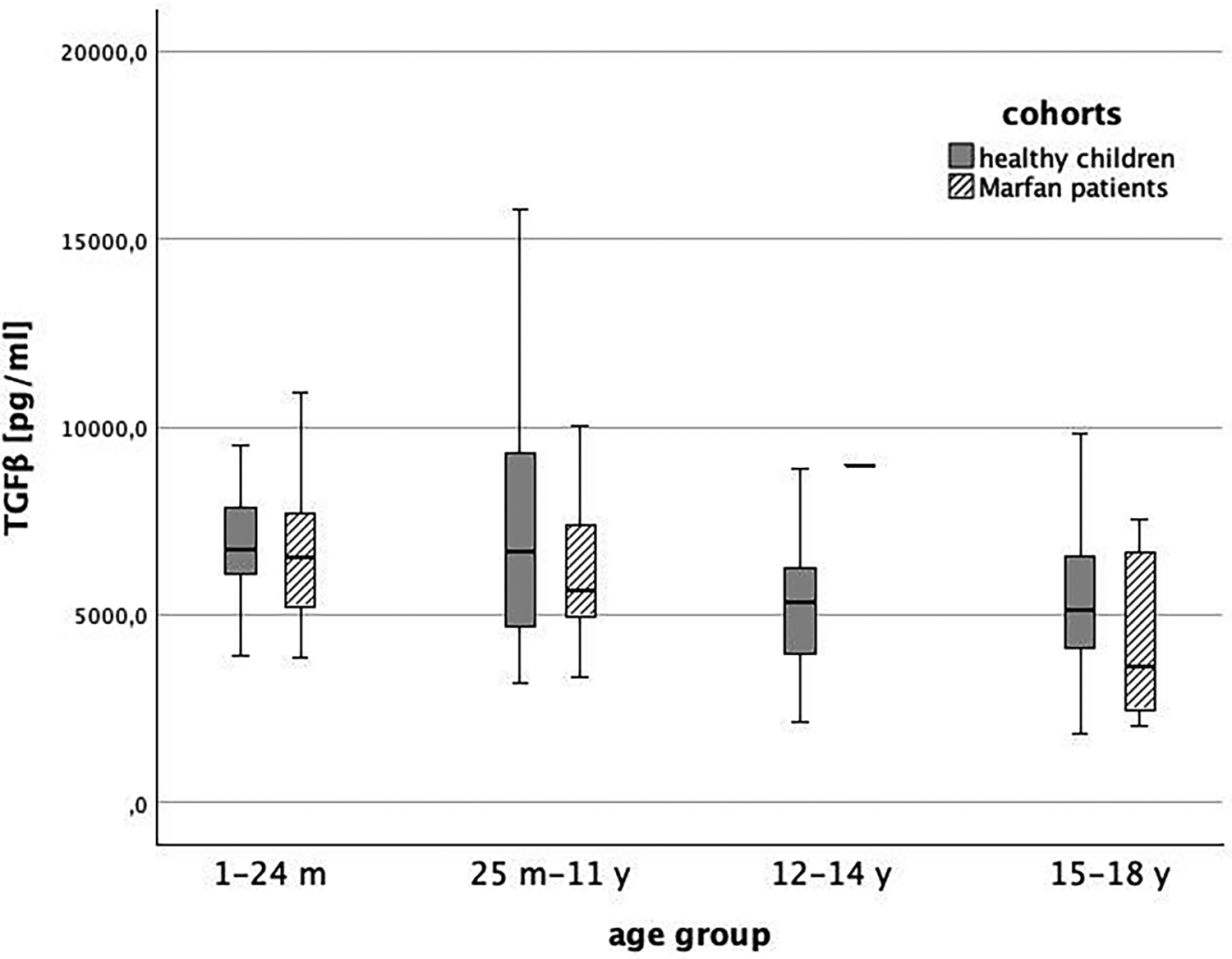
TGFβ level in healthy children (grey) and children with Marfan syndrome (striped) indifferent age groups (1 = 1-24 months, 2 = 25 months-11 years, 3 = 12-14 years, 4 = 15-18 years, *p* > 0.0125).

Baseline TGFβ serum levels were 6,137 (95% CI: 5,360; 6,914) pg/ml in MFS patients without medication and 6,526 (95% CI: 6,027; 7,026) pg/ml in healthy children.. We could not show a significant difference between both groups (*p* > 0.0125). In Figures 3, 4, we illustrate the comparison of TGFβ serum levels between MFS patients and healthy children by means of boxplots.

**Figure 5:**
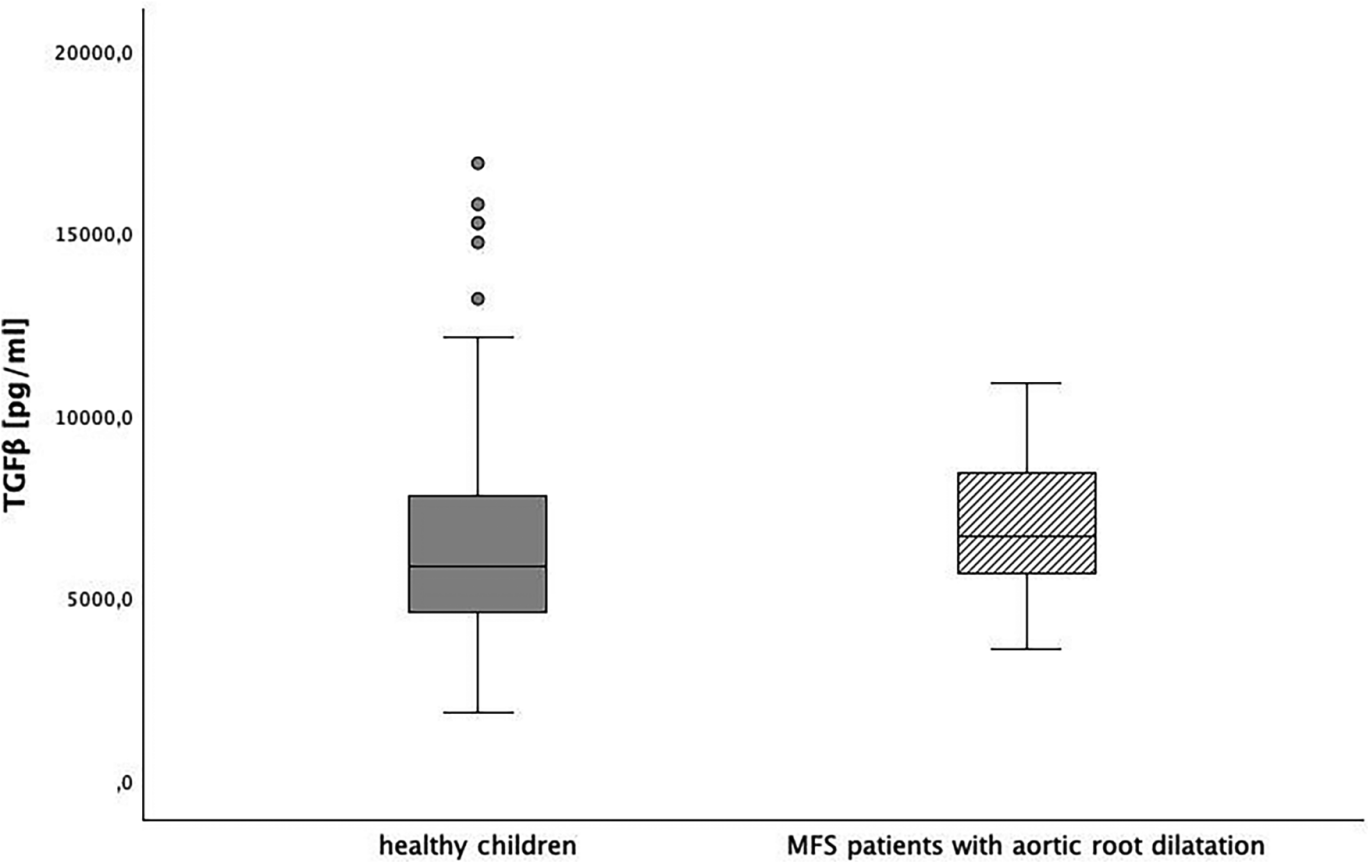
TGFβ level in healthy children and children with Marfan syndrome (MFS) with aortic root dilatation, *p* = 0.028.

We also compared TGFβ levels between children without chronic disease [6,517 (95%-CI: 6,014; 7,020) pg/ml] and pediatric Marfan patients with aortic root dilatation [6,986 (95%-CI: 5,633; 8,339) pg/ml]. Again, there was no significant difference (*p* = 0.28) (Figure 5).

**Figure 6:**
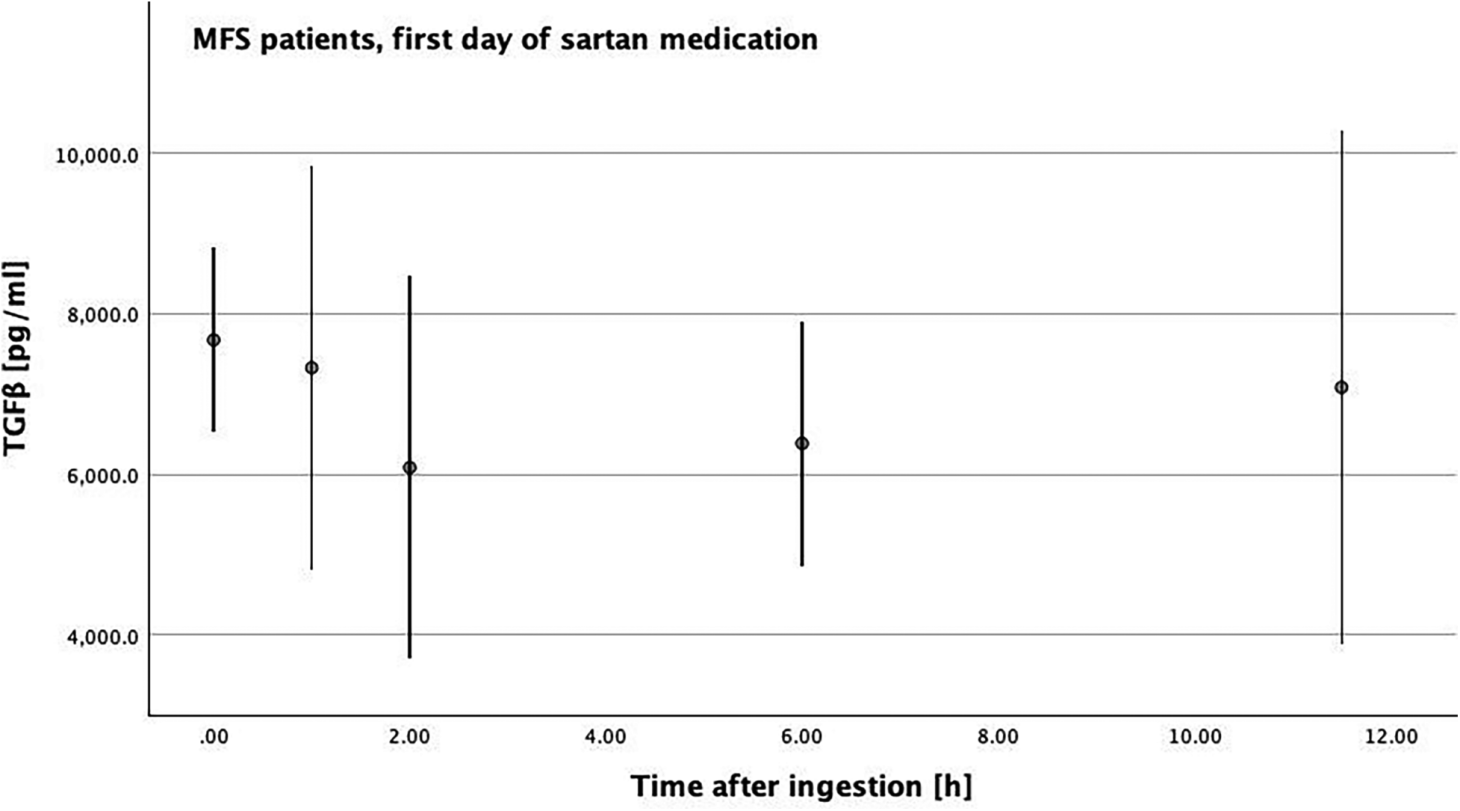
TGFβ level development 1, 2, 6, and 12 h after valsartan intake.

In 11 patients (5.5 ± 3.8 y, male: 72.7%), medical treatment was indicated. After administration of Valsartan, the lowest TGFβ serum levels were reached after 6 h, with an average reduction of 1,288 pg/ml (95% CI: 85;2,491, *p* < 0.0125), followed by a return toward baseline at the 11.5-hour mark [delta from baseline −590 pg/ml (95% CI: −2,932;1,751, *p* > 0.0125), Figure 6]. Measuring TGFβ at random times 12 months later did not show a TGFβ level decrease in comparison to baseline. There was no significant change in the z-score of the sinus valsalvae of the Marfan patients during the short observation period of 1 year. In our patient collective, no relevant difference in TGFβ levels between haploinsufficient or dominant-negative gene variants could be detected at neither baseline nor after ARB treatment.

## Discussion

Our TiGer For Kids study is, so far, the largest study to examine TGFβ levels in healthy children and in comparison with MFS children.

We firstly investigated serum TGFβ levels in children without chronic disease and children with MFS. Secondly, we analyzed the role of the biomarker TGFβ with regard to effectiveness and possible therapy monitoring in Marfan patients on the basis of TGFβ measurements.

### TGFβ level in children without chronic disease

In our study, we examined the serum TGFβ level in healthy children. TGFβ plays an essential role in many processes of life and is a physiological cell parameter in the context of cell growth and differentiation, apoptosis, embryogenesis, and development, but it is also relevant in immune processes, wound healing, inflammation, and neoplasia (13). Most tissue types possess TGFβ receptors (14). Thus, TGFβ plays a role not only in the regulation of the cardiovascular system but also in the development of lung diseases, tumors, and inflammatory processes. Even the physical activity before the measurement influences the resulting value significantly (15). Only the combination of structurally altered microfibril ECM with dysregulation of TGFβ causes the symptoms of Marfan syndrome. Measuring TGFβ levels in healthy children to detect a possible connective tissue weakness as a screening tool is therefore not useful. Nevertheless, TGFβ normal values in a healthy collective would be helpful to classify the TGFβ level in diseased children. Our measurements showed a decreasing TGFβ level with age in healthy children, especially at the beginning of the second decade of life. Neither sex nor increased inflammatory or renal retention parameters had any influence on the TGFβ level. There are currently a few smaller studies looking at TGFβ norm levels overall and those in childhood. Okamoto et al. compared the TGFβ levels of 55 healthy children with 44 healthy adults, showing significantly lower levels in adulthood, consistent with our findings (16). Rosensweig et al. published similar results of decreasing TGFβ levels from infancy through adolescence into adulthood (17). Our collective consisted of children who presented to the pediatric clinic without chronic and systemic diseases, especially without cardiovascular disease. However, we were unable to standardize the physiological processes that also influence TGFβ levels. This resulted in a relatively heterogeneous collective. We have discussed the study limits in this regard in the limitations section.

### TGFβ level in pediatric Marfan patients

Apart from abnormal fibrillin 1, excessive TGFβ activation is essential for the pathogenesis of MFS (3, 18, 19). Thereby, elevated TGFβ levels have been proven in different compartments of the body. Elevated TGFβ levels in Marfan patients have been detected in aortic tissue samples and in the peripheral blood of adult Marfan patients at the same time (7). Thus, TGFβ as a biomarker for aortic dilatation in MFS patients could be suggested. In our pediatric collective, the levels of TGFβ in the serum of Marfan patients did not differ significantly from those children without chronic disease. The comparison of healthy children with Marfan patients with aortic root dilatation also showed no significant difference. This correlates with previous data where TGFβ was also not increased compared to healthy controls (20). In other studies, particularly involving adult patients with aortopathies and especially MFS, an increased TGFβ level could be measured compared to the healthy collective (8, 11, 21). The prerequisites for the inclusion of healthy comparison groups are not always defined in detail in the various publications on adult patients. As already mentioned, TGFβ level is a biomarker that is influenced in many ways and could also possibly be subject to significant diurnal fluctuations, especially during growth. Therefore, the selection of our healthy comparison cohort might have been too inconsistent. Again, so far, TGFβ is no sufficient parameter for screening for connective tissue disease.

### TGFβ level in pediatric Marfan patients with ARB treatment

Beta-blockers have been the gold standard in the treatment of MFS for almost 50 years (19, 22). Accordingly, the experience gained by physicians and patients with the medication is great. In addition to the hemodynamic effect with the reduction of heart rate and lowering of blood pressure, they cause an increased distensibility of the aortic wall and a reduced pulse wave velocity. This effectively inhibits aortic root dilatation (23, 24). Beta-blockers are also effective in the pediatric population and are accordingly recommended and used at an early age (24–26).

Since an inhibition of the excessive activity of TGFβ in Marfan disease could be demonstrated by the use of ARBs (angiotensin II receptor type 1 blockers) in pregnant mice and is presumable in humans too, their use appears rational in young patients (5, 9, 25). For more than 15 years, studies investigating the perfect treatment have dominated the scientific world of MFS. A superior effect of ARBs in comparison to beta-blockers has not yet been proven (27). However, an equivalent effect has been demonstrated in numerous large patient groups (27–30). In addition, ARBs have been shown to have a low side-effect profile in childhood (31). Moreover, there are a number of studies that tend to recommend ARBs in Marfan patients, whereas studies exclusively recommending beta-blockers are lacking (31–33). Therapy with sartans has been shown to be effective in adulthood, particularly in patients with haploinsufficient gene variants (10, 21). Our measurements showed no relevant difference between the TGFβ levels of haploinsufficient and dominant-negative patients either without or with therapy. We measured pediatric TGFβ levels before and during therapy with ARBs to assess the potential of TGFβ levels for therapy monitoring. Patients with sartan treatment showed an effective TGFβ suppression during the day, with the lowest level of six hours after ingestion and a renewed rise right before the second dose. Concerning TGFβ suppression, our results suggest that therapy with sartans in childhood seems to be reasonable, even though the z-score of the aortic diameter did not change significantly during the short period of observation. The next step is to correlate the TGFβ level reduction after ARBs use with the reduction in aortic root diameter over a longer period of time. Starting ARB therapy before the development of an aortic aneurysm does not appear to be indicated yet. Cook et al. investigated and explained the contrary TGFβ effect in Marfan patients. The early use of TGFβ inhibition in mice without aortic aneurysm even increased the risk of developing aortic dilatation or dissection. Only after aortic dilatation occurs does TGFβ inhibition reduce the progression of the disease. Accordingly, therapy should be started as early as possible after the occurrence of the aortic aneurysm (34). In line with our results, it seems to be reasonable to apply sartans twice daily for a constant suppression. The assumption remains that ARBs could even establish themselves as a superior therapy due to their effect that goes far beyond the antihypertensive effect. Based on our data and data published since then, TGFβ is neither suitable as a screening parameter for MFS nor as a biomarker for the progression of aortic aneurysms. Other cytokines from the TGFβ pathway such as IL11 are being investigated in order to find laboratory chemical, measurable parameters to perhaps one day enable a prognostic assessment of the disease (35).

### Study limitations

Overall,the TGFβ-levels are broadly distributed not only in the children without chronic disease but also in the MFS patients. Of course, a standardized examination and timing would have produced clearer results. However, in our clinical practice, this could not be realized in both patient groups. Especially, the inclusion of healthy children with blood sampling was difficult. According to the ethics vote, blood sampling was only allowed if it was already indicated for other clinical reasons or a peripheral indwelling cannula was inserted. Thus, the timing and physiological fluctuations and prerequisites of the patients were different. Furthermore, pediatric Marfan patients received a TGFβ level measurement in the context of a routine blood collection whenever and under which conditions the presentation in our consultation took place. We accepted this for obtaining the first TGFβ measurements from the children. A clear statement that the TGFβ level differs or not between non-diseased patients and patients with MFS can therefore not be conclusively assessed by our study.

In addition to the broad distribution of the TGFβ values, it is also noticeable that in individual patients, the valley level of TGFβ before the next drug administration exceeds the initial level (Figure 6). We also explain this by the pronounced susceptibility of the TGFβ level to disturbances, in particular by physiological factors (e.g., physical stress, food intake), which were not standardized in this study.

Furthermore, although our healthy population includes children without chronic and cardiac diseases, it is known that infections or trauma can also influence the TGFβ level. However, no correlation with increased TGFβ could be found with analysis of the CRP value.

We did not find a clear TGFβ reduction after a half year or a year of ARB treatment. The TGFβ measurement after 6 and 12 months again was carried out at the time of the presentation in our Marfan consultation and not in a clear temporal relation to the ARB intake. In addition, as explained previously, the TGFβ level is very susceptible to interference and fluctuates over the course of the day. Accordingly, the follow-up level cannot be classified under these non-standardized conditions and is not helpful for the assessment of an effective therapy. Possibly, valley level measurements during the course of therapy could provide further information and improve the usefulness of this biomarker in therapy monitoring. Our aim was to measure circulating TGFβ in order to establish an uncomplicated parameter for everyday clinical use. However, it is known that the activated TGFβ level is particularly relevant and that it is expressed in the affected tissue. We did not take and examine any tissue samples in our pediatric population.

In our study, we have measured the total TGFβ level in serum, as in previous studies of adult Marfan patients. However, it is now known that serum contains high concentrations of platelet-derived TGFβ1, which is released during blood clotting. However, the free, active TGFβ component appears to be particularly relevant to the disease (36). Future studies should possibly determine the total level and the free TGFβ in plasma.

## Conclusion

This study is so far the largest examination of serum TGFβ levels in children. We conclude that, first, TGFβ decreases with age, especially at the reach of puberty. Second, TGFβ level does not differ significantly between children without chronic disease and those with MFS. The screening of TGFβ levels to recognize a connective tissue disease does not appear to make sense. Third, sartan intake suppresses TGFβ level relevantly during the day with a rise again right before the second dose.

## Data Availability

The raw data supporting the conclusions of this article will be made available by the authors, without undue reservation.
